# Antidiabetic Activity of Mung Bean or *Vigna radiata (L.)* Wilczek Seeds in Alloxan-Induced Diabetic Mice

**DOI:** 10.1155/2022/6990263

**Published:** 2022-10-26

**Authors:** Yosef Eshetie Amare, Kassahun Dires, Tsegahun Asfaw

**Affiliations:** ^1^Department of Biomedical Sciences, Asrat Woldeyes Health Science Campus, Debre Berhan University, Debre Berhan, Ethiopia; ^2^Department of Pharmacy, Asrat Woldeyes Health Science Campus, Debre Berhan University, Debre Berhan, Ethiopia; ^3^Department of Medical Laboratory Sciences, Asrat Woldeyes Health Science Campus, Debre Berhan University, Debre Berhan, Ethiopia

## Abstract

**Introduction:**

Despite the development of oral hypoglycemic medications, diabetes and its associated complications continue to be significant clinical issues. The purpose of this study was to examine the antidiabetic effects of *Vigna radiata* (L.) Wilczek seeds in mice that had been given alloxan to cause diabetes.

**Methods:**

In Swiss albino mice, diabetes was brought on by a single intraperitoneal injection of the drug alloxan (150 mg/kg). For 14 days, glibenclamide (5 mg/kg) and methanol extract of *V. radiata* seeds (100, 200, and 400 mg/kg) were given orally. Following oral administration of *V. radiata* to mice, the blood glucose levels (BGL) and body weight were measured at 7 and 14 days. The mice were sacrificed at the end of the trial, and blood samples were taken for the evaluation of insulin, glycated hemoglobin, aspartate aminotransferase (AST), alanine aminotransferase (ALT), high-density lipoprotein (HDL), total cholesterol (TC), and triglyceride (TG) levels. It was determined how much glycogen was present in the liver. Additionally, the total phenolic and flavonoid contents of *V. radiata* were determined, along with the in vitro DPPH (2, 2 diphenyl-1-picrylhrazyl) free radical-scavenging activity. *P* < 0.05 was chosen as the cutoff for statistical significance.

**Results:**

Following oral administration of *V. radiata* for 14 days, diabetic mice's BGL and bad cholesterol (TC and TG) levels significantly decreased, while HDL levels increased. Treatment with *V. radiata* significantly decreased the levels of AST, ALT, and glycated hemoglobin when compared with diabetes control. On the other hand, it raised insulin levels and the amount of liver glycogen. *V. radiata* underwent phytochemical analysis, which identified the presence of tannins, saponins, phenols, alkaloids, terpenoids, steroids, flavonoids, and glycosides. Per gram of *V. radiata* seed extract, the total phenolic content was 43.12 ± 3.14 mg of gallic acid equivalents, while the total flavonoid content was 38.35 ± 2.6 mg of quercetin equivalents. Ascorbic acid was shown to have an IC_50_ value of 18.64 *µ*g/ml during a DPPH-scavenging assay, while *V. radiata* had an IC_50_ value of 73.35 *µ*g/ml.

**Conclusion:**

According to the findings of the current study, the methanolic extract of the seeds from the plant *V. radiata* possesses significant antidiabetic characteristics that are on par with those of the commonly used drug glibenclamide. Hence, *V. radiata* seems to be effective as a natural antidiabetic.

## 1. Introduction

Elevated blood glucose levels (BGL) brought on by deficiencies in insulin secretion, action, or both characterize the metabolic disease known as diabetes mellitus (DM). In 2021, 536.6 million individuals in the world were estimated to have diabetes; by 2045, that number was projected to rise to 783.2 million. Costs associated with treating diabetes worldwide were estimated at 966 billion USD in 2021 and are expected to rise to 1,054 billion USD by 2045 [1]. It is now a global public health issue that can cause both macrovascular and microvascular problems [2]. Diabetes already poses a serious threat to global health and places a heavy strain on the sub-Saharan region; this is only anticipated to get worse. Access to diagnosis and treatment is difficult for many patients with DM, which raises mortality and increases the likelihood of complications [3]. Diet, exercise, and contemporary medications like insulin and oral hypoglycemic medicine are used to manage DM [4].

Modern diabetes medications have severe side effects such as edema, lactic acidosis, weight gain, and hypoglycemia [5]. This has led to a continuous search for safer and more potent diabetes medications. Medicinal plants have been used as a source of medicine all across the world. A whopping 80–85% of people worldwide use herbal extracts to cure illnesses [6]. Traditional methods of managing diabetes have also included the use of medicinal herbs, which are seen as being relatively inexpensive, less poisonous, and having few to no adverse effects [7]. From medicinal plants, several active ingredients have been extracted for usage directly as medications, lead compounds, or pharmacological agents. One such historic instance is the use of metformin, which was isolated from *Galega officinalis*, to treat diabetes in medieval Europe [8].

The mung bean or *Vigna radiata* (L.) Wilczek is also known as Oregon pea, chickasano pea, chiroko, and just plain mung. According to reports, the crop was first grown in India and has since spread to South and East Asia, East and Central Africa, the West Indies, and the United States. Low-altitude mung bean cultivation ranges from sea level to around 2000 m, typically as a dry land crop. It grows most successfully in well-loam soil with evenly spaced rains. It is resistant to drought but is vulnerable to waterlogging [9]. *V. radiata* has a high isoflavone content, according to previous studies [10, 11]. Previous studies have shown that isoflavones derived from plants tend to reduce blood sugar levels [12–14]. Studies on the phytochemistry of *V. radiata* have discovered flavonoids, alkaloids, phenolic compounds, and terpenoids [15, 16]. The presence of secondary metabolites such as flavonoids, terpenoids, alkaloids, and phenolic chemicals is primarily responsible for the antidiabetic effect of some medicinal plants [13, 17–21]. *V. radiata* has also been utilized as a dietary food for the treatment of DM because it has a low glycemic index and high protein and fiber content [22, 23].

The primary pathophysiology of DM and its consequences is the production of oxidative stress [13, 24, 25]. Through the reduction of oxidative stress, antioxidants are able to treat diabetes and its associated consequences, according to many research [13, 25, 26]. *V. radiata* may be a target in the search for novel potential medications for the treatment and prevention of DM because prior research on the plant has demonstrated that it has excellent antioxidant activity [10, 27, 28]. Additionally, studies have shown that mung beans can reduce hyperlipemia and hypertension as well as prevent cancer and melanogenesis [22]. Mung bean seeds are utilized in traditional medicine in the majority of Asian nations, Africa, and South America to treat diseases like DM, heat stroke, and others [22]. However, there has not been much research to support the antidiabetic properties of *V. radiata.* The purpose of the current study was to assess the antihyperglycemic effect of a methanolic extract of *V. radiata* (Mung Bean) seeds in diabetic mice.

## 2. Materials and Methods

### 2.1. Drugs and Chemicals

The following medications, substances, and equipment were utilized in this study: alloxan (Sigma–Aldrich, Germany), methanol absolute (Nice Chemicals, India), glibenclamide (Julphar Pharmaceuticals, Ethiopia), normal saline (Addis Pharmaceutical factory, Ethiopia), 40% glucose solution (Addis Pharmaceutical factory, Ethiopia), PRODIGY® blood glucose meter, and strips (Ok Biotech Co., Ltd., Taiwan), Mindray BS-240 clinical chemistry analyzer (Shenzhen Mindray Bio-Medical Electronics Co., Ltd., China). Analytical-grade chemicals were employed throughout.

### 2.2. Plant Material and Extraction

**Figure 1:**
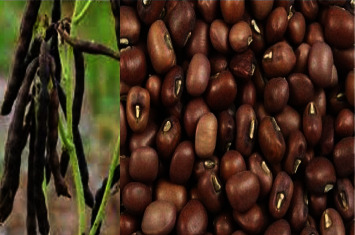
*V. radiata* seeds.

The dried *V. radiata* seed (10 kg) was gathered in the Ankober district, North Shoa Zone, Amhara region, Ethiopia (Figure 1). Then, taxonomic identification and authentication were completed. The plant specimen has been given the voucher number ATA0002 and was stored in the Addis Ababa University Herbarium. 500 g of dried and ground seeds were extracted using the soxhlet technique with 100 mL of methanol for two hours, yielding 150 g of crude extract. The bulk of the crude extract was weighed and kept at −4°C until usage.

### 2.3. Phytochemical Screening Tests

Standard methods have been used to detect phytochemical components [29–31].

The application of Salkowski's technique allowed for the detection of terpenoids. 100 mg of extract was dissolved in 5 ml of distilled water. 2 ml of chloroform and 3 ml of strong sulfuric acid were added to the solution. At the interface, a layer of reddish-brown color developed, indicating the presence of terpenoids.

Braemer's test was used to identify tannins. 0.25 g of the crude extract was added to a test tube along with 10 mL of distilled water and filter paper (Whatman No. 1). To the filtrate, a few drops of 2% ferric chloride were added. To determine whether tannins were present, the filtrate was examined for any green precipitation.

To find saponins, the Froth test was applied. The test tube was filled with 0.25 g of crude extract and 5 milliliters of distilled water. The solution was vigorously agitated for two minutes during which time a strong, enduring froth formed. The presence of saponins is indicated by the development of foam.

Borntrager's test was used to examine for the presence of anthraquinones. Following the dissolution of 3 ml of the plant extract in 3 ml of benzene, Whatman No. 1 filter paper was used for the filtering process. 10% ammonium hydroxide was then added at a volume of 2 ml. Anthraquinones were present in the extract as shown by the development of purple rings.

Liebermann-Burchardt test was employed to find the presence of steroids. In a test tube, 0.5 g of the methanolic crude extract was dissolved in 2 mL of distilled water. Then, 2 mL of sulfuric acid and chloroform were added to the mixture. Steroids were present because of the creation of a red tint in the chloroform layer's lower layer.

To identify the presence of alkaloids, Wagner's test was used. 10 mg of the crude extract was combined with three drops of Wagner's reagent and dissolved in distilled water. Alkaloids can be detected by their reddish-brown color development.

For finding phenolic compounds, the ferric chloride test was employed. The dissolved 10 mg crude extract in 1 ml of distilled water was then mixed with 0.5 ml of neutral 5% ferric chloride solution. Indicative of the presence of phenolic compounds is the creation of a blue-green tint.

The test for flavonoids was conducted using NaOH. 2 ml of distilled water was used to dissolve almost 0.3 g of crude extract. The solution was supplemented with three drops of a 20% NaOH solution. When three drops of 20% hydrochloric acid are added to the solution, the yellow hue changes to colorless, indicating the presence of flavonoids.

Keller–Kliani test was used to find glycosides. 0.5 g of dried crude extract was dissolved in 20 ml of distilled water in a test tube. After 24 hours, the solution was filtered using Whatman No. 1 filter paper. Two milliliters of concentrated glacial acetic acid and two drops of 0.1% ferric chloride solution were added to five milliliters of the filtrate. A test tube containing 1 ml of concentrated sulfuric acid was filled with the combination. The presence of glycosides in the extract is shown by the brown ring development at the contact.

### 2.4. Determination of Total Phenolic and Flavonoid Contents in *V. radiata* Extract

To determine total phenolic content, a Folin-Ciocalteu reagent was utilized. 400 *µ*ml of Folin-Ciocalteu reagent, 1.5 ml of 20% sodium carbonate, and 200 *µ*g/ml of extract were combined. The mixture was well shaken before adding 10 ml of distilled water. The mixture's absorbance at 765 nm was measured after two hours. The amount of phenol overall in the *V. radiata* extract was calculated using a gallic acid standard curve and was represented as mg of a gallic acid equivalent per gram of the plant extract.

Aluminum trichloride was combined with 1 ml of methanolic extract (200 *µ*g/ml) in methanol (20 mg/ml). A drop of acetic acid was added, and 20 ml of methanol was used to dilute it. Prior to determining the absorbance, the solution was filtered using Whatman filter paper No. 1. After 45 minutes, the absorbance was then measured at 415 nm against a blank. The standard quercetin curve was used to calculate the total flavonoid content of *V. radiata*, which was then expressed as mg of quercetin equivalent per gram of the plant extract.

### 2.5. Antioxidant Activity of *V. radiata* Using DPPH Free Radical-Scavenging Assay

To evaluate *V. radiata's* antioxidant capacity, the DPPH (2, 2 diphenyl-1-picrylhrazyl) free radical-scavenging technique was used. Ascorbic acid was utilized as a control substance under the assumption that it completely scavenges free radicals. In test tubes containing 200 *µ*ml of methanol solution of the plant extracts at various concentrations, 2.8 ml of DPPH solution (45 *µ*g/ml) was added right away. After being properly blended, the solution was left at room temperature for 30 minutes. Finally, using a blank of methanol solution, the absorbance was measured at 517 nm. Using the following formula, the fraction of DPPH free radicals that were scavenged was determined:

(1) $$\begin{array}{c} \left\{\frac{\left(A_{0}-A_{1}\right)}{A_{0}}\right\}\times 100\text{,} \end{array}$$

where *A*_0_ = absorbance of the control/standard; *A*_1_ = absorbance of the extract. Following that, the percentage (%) of inhibition was plotted against the log concentration, and the IC_50_ was determined from the graph.

### 2.6. Experimental Animals

Swiss albino mice that were eight to twelve weeks old and weighed between 25 and 30 grams were used in this study. The acute toxicity research, oral glucose tolerance test, and antidiabetic test all utilized a total of 71 mice. They were bought from the Public Health Institute in Ethiopia. The animals were housed in the Department of Pharmacology at Addis Ababa University in polypropylene cages. Prior to the trial, the animals experienced a seven-day acclimatization phase under usual laboratory conditions, including a regular meal, unlimited access to water, and a light and dark cycle. The ARRIVE guidelines were followed in conducting this investigation. The experiment was carried out in accordance with the protocol for the care and use of experimental animals [32].

### 2.7. Oral Acute Toxicity Study

Based on the Organization for Economic Co-operation and Development's (OECD) guideline limit test requirements, an acute oral toxicity test was conducted [33]. On the first day of the test, a female Swiss albino mouse that had been fasting for three hours was administered orally 2000 mg/kg of crude extract. After being closely monitored for 24 hours for behavioral and/or physical changes, the first mouse did not perish. The following four female mice were given a single dosage of 2000 mg/kg *V. radiata* seed extract after a 4-hour fast. As a result, close monitoring was maintained for any side effects until the end of the 14-day period.

### 2.8. Oral Glucose Tolerance Test (OGTT) in Mice with Normoglycemia

The mice were divided into five groups randomly after a 16-hour overnight fast (six mice per group). Then, in accordance with their grouping, animals received treatment with normal saline, plant extract, or glibenclamide. Each mouse received 2.5 g/kg of oral glucose solution (40% w/v) after each treatment lasted for 30 minutes [34–36]. As a baseline measurement, the BGL of each mouse was measured at 0 minutes, followed by measurements at 30, 60, and 120 minutes after an oral glucose load [34, 35, 37].

1. Normal Control (Group NC, 10 ml/kg NS, *n* = 6)
2. Standard Drug (Group SD, Glibenclamide (5 mg/kg), *n* = 6)
3. Extract (Group VR100, 100 mg/kg, *n* = 6)
4. Extract (Group VR200, 200 mg/kg, *n* = 6)
5. Extract (Group VR400, 400 mg/kg, *n* = 6)

### 2.9. Induction of Diabetes

Alloxan (2, 4, 5, 6-tetraoxypyrimidine; 5, 6-dioxyuracil) was administered to each mouse to induce diabetes. Before receiving alloxan, the animals went on a 16-hour fast [35]. Fresh solutions of alloxan were given intraperitoneally to each mouse at a dose of 150 mg/kg after being dissolved with the appropriate volume of normal saline (0.9%) [38–40]. Following the administration of alloxan, animals were examined for diabetes 72 hours later, and those with fasting BGL higher than 200 mg/dl were designated as diabetic mice for the study [35, 39, 41, 42].

### 2.10. Grouping and Dosing of Experimental Animals

Thirty diabetic mice were divided into five groups of six animals each at random. The normal control group consisted of an age-matched group of healthy mice. After being dissolved in normal saline at a volume of no more than 10 ml/kg body weight of the specific animal, the conventional medication and plant extract were delivered by gastric tube [33]. Since humans often consume plant material orally, the study employed this method of treatment [22]. The standard antihyperglycemic medication was glibenclamide (5 mg/kg), which was chosen based on prior trials [34, 41].

1. Normal Control (Group NC, 10 ml/kg NS, *n* = 6)
2. Diabetic Control (Group DC, 10 ml/kg NS, *n* = 6)
3. Diabetic + Standard Drug (Group DS, Glibenclamide (5 mg/kg), *n* = 6)
4. Diabetic + Extract (Group VR100, 100 mg/kg, *n* = 6)
5. Diabetic + Extract (Group VR200, 200 mg/kg, *n* = 6)
6. Diabetic + Extract (Group VR400, 400 mg/kg, *n* = 6)

### 2.11. Testing the Antihyperglycemic Activity of *V. radiata* Extract

According to their assigned groups, mice received glibenclamide, plant extract, and normal saline in regular intervals once daily for 14 days. Prior to the start of treatment, on day 0, the mouse BGL was assessed. Throughout the course of treatment, measurements were also taken on days 7 and 14 [43]. Each mouse's tail vein was used to collect blood samples using an aseptic technique. We measured the BGL using a PRODIGY® blood glucose meter. In every instance, the BGL was measured in triplicate before being averaged.

### 2.12. Assessing Body Weight Changes and Analysis of Lipid Profile

The body weights of mice were measured just before the initiation of treatment, on the 7^th^ and 14^th^ days of treatment. On the fifteenth day, sodium pentobarbitone (150 mg/kg) was administered intraperitoneally to sacrifice the mice. Blood samples were subsequently taken using sterile gel tubes by heart puncture [35, 43]. The blood samples were centrifuged after being kept at room temperature for two hours. Then, using a Mindray BS-240 clinical chemistry analyzer (Shenzhen Mindray Bio-Medical Electronics Co., Ltd., China), serum levels of high-density lipoprotein (HDL), total cholesterol (TC), and triglyceride (TG) were determined.

### 2.13. Determination of Liver Glycogen, Aspartate Aminotransferase (AST), and Alanine Aminotransferase (ALT) Levels

A little amount of each mouse's liver was extracted using o-toluidine-glucose, followed by coupling with trichloroacetic acid (TCA), precipitation with alcohol, and hydrolysis. Finally, a UV spectrophotometer was used to estimate glycogen [44]. Serum was separated from the blood samples by centrifugation at 4000 rpm for 20 minutes, and the serum was then kept at −20°C for UV Spectrophotometric analysis of AST and ALT levels (Shimadzu UV-1200, Tokyo, Japan) by employing diagnostic wet reagent kits in accordance with the manufacturer's instructions (Centronic GmbH Germany and Crescent Diagnostic Kits) [45].

### 2.14. Determination of Insulin and Glycated Hemoglobin

The Nayak and Pattabiraman method [44] was used to estimate glycated hemoglobin. Using an Insulin-1 ELISA kit, the amount of insulin was tested (Sigma–Aldrich Germany). The color created was measured at 450 nm after 30 minutes.

### 2.15. Data Analysis

IBM SPSS statistics for windows, version 21 (IBM Corp., Armonk, N. Y., USA) was used to enter, summarize, and analyze the data. Using mixed-design ANOVA, the mean and standard error were computed and the association between and within groups was determined. The *P*-value of less than 0.05 was used to reflect the results of the statistical significance level.

## 3. Results

### 3.1. Percentage Yield of the Plant Extract

It was discovered that the methanolic seed extract of *V. radiata* had a 30% (w/w) yield. At room temperature, the extract was reddish semisolid, and when kept in the refrigerator, it solidified.

### 3.2. Phytochemical Screening

As shown in Table 1, the phytochemical screening test of the methanolic extract of *V. radiata* seeds revealed the presence of terpenoids, steroids, flavonoids, glycosides, tannins, saponins, phenols, and alkaloids.

### 3.3. Total Phenolic and Flavonoid Contents

**Figure 2:**
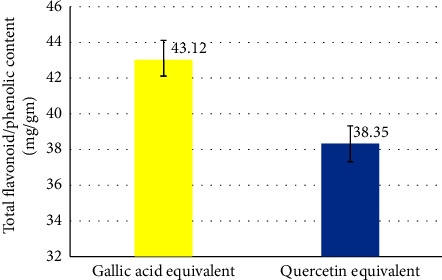
The total phenolic and flavonoid content of *V. radiata* is represented as the equivalent to gallic acid and quercetin, respectively.

Figure 2 shows that the total phenolic content per gram of *V. radiata seed* extract was 43.12 ± 3.14 mg of a gallic acid equivalent, while the total flavonoid content was 38.35 ± 2.6 mg of quercetin equivalents.

### 3.4. Effect of *V. radiata* on DPPH Free Radical-Scavenging Activity

**Figure 3:**
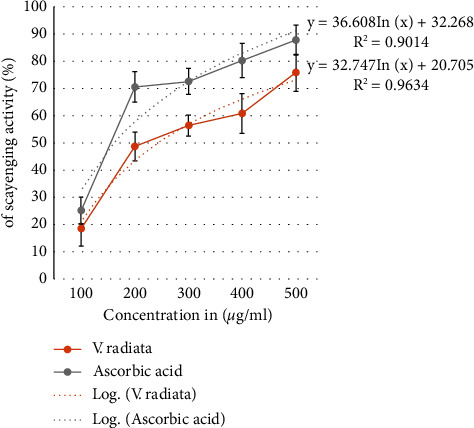
DPPH free radical-scavenging activity (%) at various concentrations *µ*g/ml of *V. radiata* extract and ascorbic acid.

The ability of a *V. radiata* extract to scavenge free radicals was evaluated using DPPH free radical-scavenging assay. A substance's IC_50_ value tells us whether it has antioxidant properties.  Figure 3 displays the results of the *V. radiata* extract's DPPH-scavenging activity. Compared to ascorbic acid, which had an IC_50_ of 18.64 *µ*g/ml, the extract had an activity for scavenging DPPH radicals with a value of 73.35 *µ*g/ml.

### 3.5. Acute Oral Toxicity Study

The methanolic seed extract of *V. radiata* was safe up to a dose level of 2000 mg/kg of body weight in mice since no mortality or symptoms of toxicity (behavioral, autonomic, neurological, or physical abnormalities) were seen in the animals during the study period. Based on the findings of an investigation into acute oral toxicity, the doses of the plant extract were established. The lower dose of 100 mg/kg was determined as half of the middle dose, the higher dose of 400 mg/kg is twice the middle dose, and the intermediate dose of 200 mg/kg is one-tenth of the limit dose (2000 mg/kg).

### 3.6. Effect of *V. radiata* on OGTT

Table 2 displays the effects of *V. radiata* extract on OGTT. Before extract delivery (0 min), the BGL of all groups did not appear to differ from one another. However, all groups experienced a substantial increase (*P* < 0.01) in BGL 30 minutes after extract administration (one hour after oral glucose loading), indicating the induction of hyperglycemia. At 60 minutes, the NC group's hyperglycemia following the glucose test had not considerably decreased, but at 120 minutes, there had been a significant change (*P* < 0.01). In contrast, compared with the glucose level at 30 minutes, all dose groups of the plant extract decreased the BGL level after 60 minutes (*P* < 0.01) and 120 minutes (*P* < 0.001). At 60 and 120 minutes, the DS group also significantly reduced hyperglycemia (*P* < 0.001). Overall, all plant extract and DS dose groups have shown the capacity to lower BGL at 120 minutes (*P* < 0.001).

### 3.7. Antihyperglycemic Activity of *V. radiata* on Alloxan-Induced Diabetic Mice

The difference in baseline BGL was not significant in any of the diabetic mouse groups (Table 3). In comparison with the diabetic control group, BGL considerably decreased in the VR200 (*P* < 0.05) and the VR400 (*P* < 0.01) treated groups on day 7 of treatment. In addition, on the 14^th^ day of treatment, BGL significantly decreased in the VR100, VR200, and VR400 treated groups (*P* < 0.01 and *P* < 0.001, respectively) compared to diabetes control. The BGL of the glibenclamide-treated group was also found to be considerably lower on the seventh (*P* < 0.01) and fourteenth (*P* < 0.001) days of therapy compared to the diabetic control group. In comparison to the glibenclamide-treated group, none of the plant extract-treated groups have consistently displayed a BGL difference that is statistically significant. Furthermore, when groups treated with plant extract were compared to one another at all time periods, no statistically significant change in BGL was found. On the seventh and fourteenth days of therapy, all *V. radiata*-treated groups displayed significantly lower BGL (*P* < 0.001) than their baseline BGL. Diabetes and normal control groups, however, did not exhibit a decline in BGL when compared to their respective baseline BGL on the seventh and fourteenth days.

### 3.8. Effect of *V. radiata* on Body Weights of Alloxan-Induced Diabetic Mice

As indicated in Table 4, there was no statistically significant difference in body weight between any of the study groups just prior to the development of diabetes. On the seventh day of treatment, only 400 mg/kg of *V. radiata* significantly (*P* < 0.05) improved body weight compared to diabetes control. However, on the 14^th^ day of treatment, body weight significantly increased with VR100 (*P* < 0.05), VR200, and the VR400 (*P* < 0.001) compared to diabetes control. On the seventh and fourteenth day of therapy, glibenclamide significantly (*P* < 0.001) enhanced the body weight loss of alloxan-induced diabetes mice compared to the diabetic control. By the 14^th^ day of treatment, the diabetic control group had significantly (*P* < 0.01) lost body weight compared to its initial body weight. The normal control group, however, showed a significant (*P* < 0.05) increase in body weight on day 14 compared to baseline.

### 3.9. Effect of *V. radiata* on Lipid Profiles of Alloxan-Induced Diabetic Mice

As shown in Table 5, in comparison to the normal control group, there were significant increases in TC and TG in the diabetic control group (*P* < 0.001) and significant decreases in HDL (*P* < 0.001). However, after fourteen days of treatment, VR100 and VR200 (*P* < 0.05), and the VR400 (*P* < 0.001), considerably enhanced the blood HDL level compared to diabetes control. All *V. radiata* doses significantly (*P* < 0.001) decreased the serum TC level. Moreover, when compared to the diabetic control, serum TG level was lowered by VR100 (*P* < 0.05), VR200 (*P* < 0.05), and the VR400 (*P* < 0.001). In addition, the glibenclamide-treated group had a substantial (*P* < 0.001) decrease in serum TC and TG and a significant (*P* < 0.01) increase in serum HDL when compared to the diabetic control group. When all plant-treated groups were compared to one another, the blood lipid profiles, including TC, TG, and HDL, were found to be barely different from one another. However, compared to the groups treated with plant extracts, the glibenclamide-treated group displayed a significant (*P* < 0.001) difference in serum levels of TC, TG, and HDL.

### 3.10. The Effects of *V. radiata* on Insulin and Glycated Hemoglobin Levels

**Figure 4:**
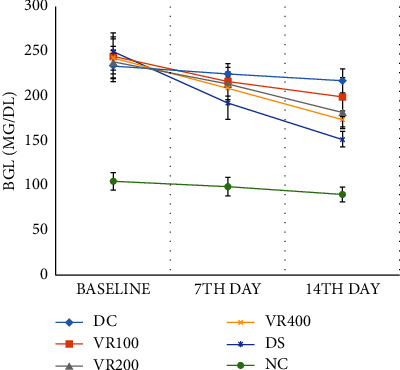
Effects of treatment of V. radiata on blood glucose level of diabetic mice. DC, diabetic control that receive 10 ml/kg normal saline; NC, normal control that receive 10 ml/kg normal saline; VR100, treatment group that receive V. radiata extract of 100 mg/kg; VR200, treatment group that receive V. radiata extract of 200 mg/kg; VR400, treatment group that receive V. radiata extract of 400 mg/kg; DS, positive control that receive glibenclamide (5 mg/kg); BGL, blood glucose level.

The effects of *V. radiata* extract on blood insulin and glycated hemoglobin levels in alloxan-induced diabetic mice are shown in Figure4. When compared with the normal control group, the insulin level in the diabetes control group was considerably lower. In contrast, all groups treated with *V. radiata* had significantly higher insulin levels than the diabetic control group (*P* < 0.001). When compared to other *V. radiata*-treated groups, the VR400 group showed a more pronounced increase in insulin levels.

The diabetes control group's glycated hemoglobin level dramatically increased. In contrast, all *V. radiata* and the glibenclamide-treated groups showed considerably lower levels of glycated hemoglobin compared to the diabetic control group. *V. radiata* had dose-dependent effects on glycated hemoglobin.

### 3.11. The Effects of *V. radiata* on Liver Glycogen, AST, and ALT Levels

**Figure 5:**
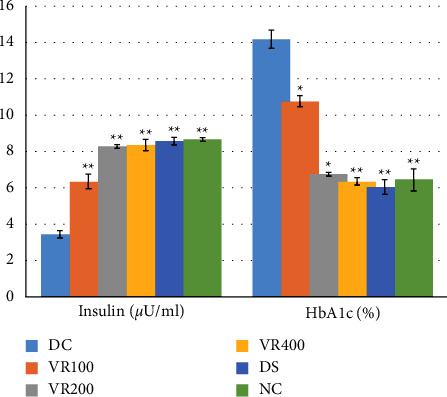
Effects of V. radiata on Insulin and glycated hemoglobin in diabetic mice. The comparison is between DC and other treatment groups. DC, diabetic control that receive 10 ml/kg normal saline; NC, normal control that receive 10 ml/kg normal saline; VR100, treatment group that receive V. radiata extract of 100 mg/kg; VR200, treatment group that receive V. radiata extract of 200 mg/kg; VR400, treatment group that receive V. radiata extract of 400 mg/kg; DS, positive control that receive glibenclamide (5 mg/kg). ^∗^*p*<0.01, ^∗∗^*p*<0.001. HbA1c, glycated hemoglobin.

The DS, VR100, VR200, and VR400 had lower liver glycogen levels than the healthy control group. As demonstrated in Figure 5, the level of glycogen content in diabetic mice treated with glibenclamide and plant extract was considerably (*P* < 0.05) higher than in the DC group. The group DC mice had considerably higher AST and ALT levels. All treatment groups that received extract exhibited a decrease in AST and ALT levels that were comparable to group DS.

## 4. Discussion

One of the largest global health crises of this generation is diabetes [46]. Modern DM medications now have issues with cost, effectiveness, and safety. The comorbid population of diabetes and dyslipidemia should receive special attention for new medications with broad-spectrum antidiabetic action and acceptable safety. Exploring plant derivative chemicals for DM is an amazing study field because they are considered secure, efficient, accessible, and cost-effective [17, 43]. The goal of the current study was to evaluate the antidiabetic effects of a methanolic extract of *V. radiata* seeds in mice that had been inflicted with diabetes using alloxan. A substance known to cause diabetes, alloxan enters the beta cells of the islets of Langerhans via the GLUT2 transporter, where it inhibits glucokinase and produces reactive oxygen species (ROS) [47, 48]. Mice that receive alloxan intraperitoneally at a dose of 150 mg/kg may experience chronic hyperglycemia [39, 48]. Similar results were found in our investigation, where the diabetic control group's BGL did not alter significantly during the two-week study.

According to the current study, 14 days of oral administration of *V. radiata* seeds methanolic extract preserved the life of diabetic mice and resulted in a significant decrease in blood glucose, lipids, AST, ALT, and glycated hemoglobin levels. It also recovered body weight, insulin, and liver glycogen levels.

**Figure 6:**
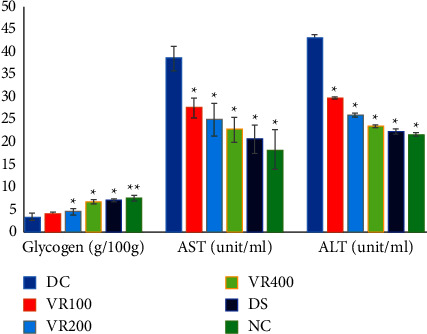
The effects of V. radiata on the levels of liver glycogen, AST, and ALT in diabetic mice. The comparison is between DC and other treatment groups. DC, diabetic control that receive 10 ml/kg normal saline; NC, normal control that receive 10 ml/kg normal saline; VR100, treatment group that receive V. radiata extract of 100 mg/kg; VR200, treatment group that receive V. radiata extract of 200 mg/kg; VR400, treatment group that receive V. radiata extract of 400 mg/kg; DS, positive control that receive glibenclamide (5 mg/kg). ^∗^*p*<0.05, ^∗∗^*p*<0.01. AST, aspartate aminotransferase; ALT, alanine aminotransferase.

*V. radiata* was shown to lower increased blood sugar levels in alloxan-induced diabetic mice at all dose levels tested (100 mg/kg, 200 mg/kg, and 400 mg/kg) (Figure 6). Numerous medicinal herbs have been found to have antihyperglycemic properties and to stimulate the release of insulin [49, 50]. The significant reduction in fasting blood sugar levels caused by *V. radiata* in alloxan diabetic mice may be attributable to the stimulation of the residual pancreatic mechanism, which is most likely brought on by an increase in peripheral glucose uptake or liver glycogen synthesis and a decrease in gluconeogenesis [51]. The findings of this study indicate that after a 14-day oral therapy with the *V. radiata* extract, insulin and glycogen levels significantly rose, which may support the potential underlying mechanisms in the lowering of blood sugar levels in diabetic mice. The blood's excessive production of glucose, which then combines with blood hemoglobin to produce more glycated hemoglobin, led to a rise in the level of glycated hemoglobin in alloxan-induced diabetic mice [52]. The levels of glycated hemoglobin and blood sugar were both considerably reduced by all doses of the *V. radiata* extract in this investigation. The direct proportionality between BGL and glycated hemoglobin could be the cause of the action's potential mechanism. Glycated hemoglobin will decrease in conjunction with a decline in blood glucose, and vice versa.

Alloxan-induced diabetes is accompanied by a distinctive reduction of body weight, which is brought on by increased muscle wasting [53] and tissue protein loss [54]. The preventive effect of *V. radiata* on the structural components of the tissue may account for the increase in body weight observed in diabetic mice treated with the plant [55].

Increases in TC, TG, and a decrease in HDL are associated with hyperglycemia and are caused by an excessive mobilization of fat from adipose tissue as a result of insufficient peripheral glucose utilization [56]. According to the findings of our investigation, diabetic mice treated with *V. radiata* methanolic extract had considerably lower levels of TC and TG and higher levels of HDL. *Vigna radiata* oral administration may have improved glucose utilization and suppressed lipid mobilization responsible for the regression of diabetes condition. Additionally, the consequences might be brought on by low levels of lipolysis, which is regulated by insulin, and/or low levels of the enzymes that produce cholesterol [57]. Triglycerides are clearly independent risk factors for coronary heart disease [58], yet the majority of lipid-lowering medications do not lower TG levels. *V. radiata* dramatically reduced TG levels, although, and this impact may be attributed to an increase in endothelium-bound lipoprotein lipase, which controls how the body eliminates lipid fuels [59].

Both the AST and ALT are accurate indicators of organ damage [60]. In mice with alloxan-induced diabetes, AST and ALT levels were abnormally elevated. Methanolic extract of *V. radiata* was taken orally, and this resulted in considerably lower AST and ALT levels among the treatment groups, indicating improved liver function. A decrease in AST and ALT levels and an increase in liver glycogen content may help to explain how the *V. radiata* extract protected the liver. This study clearly shows that oral *V. radiata* administration restored the liver glycogen content, presumably as a result of an increase in insulin levels.

Antioxidants are thought to have a suppressive effect on DPPH radicals because they can donate hydrogen [61]. Activities that neutralize free radicals are crucial to preventing their harmful effects on a variety of disorders, including diabetes. Antioxidants are known to function to reduce lipid peroxidation by scavenging DPPH free radicals. According to Figure 3, the extract's DPPH-scavenging activity increased with concentration and it displayed a noteworthy capacity to neutralize free radicals. According to the results of the phytochemical analysis, the plant *V. radiata* contains tannins, saponins, phenols, alkaloids, terpenoids, steroids, flavonoids, and glycosides. Alkaloids, phenolic compounds, flavonoids, and terpenoids were also found in earlier research on the phytochemistry of *V. radiata* [15, 16]. The overall phenolic and flavonoid levels of the extract, which are likely to have contributed to the radical-scavenging activity, are correlated with antioxidant activity. It is well known that the antioxidant effects of flavonoids depend, in general, on their capacity to donate hydrogen or an electron to a free radical. Particularly important in this regard is the hydroxyl position in the molecule.

## 5. Conclusion

The results of this investigation suggested that oral administration of the methanol extract of *V. radiata* seeds has an antihyperglycemic potential comparable to the common medication glibenclamide in a dose-dependent manner. The methanol extract of *V. radiata* seeds also moderated lipid profiles, AST, ALT, and glycated hemoglobin while restoring liver glycogen and insulin levels in diabetic mice, suggesting its potential advantages in reducing some of the consequences of diabetes. To understand the molecular mechanisms of the bioactive chemicals found in *V. radiata*, more research is necessary. Furthermore, since this study lacked histopathological examinations, future research should take these viewpoints into account to fully understand the impacts of the plant.

## Figures and Tables

**Table 1:** Phytochemicals of *V. radiata* seeds methanolic extract.

Phytochemicals	Results
Tannins	+
Saponins	+
Phenols	+
Alkaloids	+
Terpenoids	+
Steroids	+
Flavonoids	+
Glycosides	+
Anthraquinones	−

Sign (−) denotes a lack of metabolite, while sign (+) denotes its presence.

**Table 2:** The effects of *V. radiata* seed methanolic extract on OGTT in mice.

Group (*n* = 6)	BGL in mg/dl			
	0 min	30 min	60 min	120 min
NC	101.3 ± 4.7	141.2 ± 8.6^a2^	128.5 ± 7.8^a2 d3^	111.5 ± 6.8^a1 b2 d3^
VR100	106.4 ± 5.4	144.3 ± 7.2^a2^	119.9 ± 5.1^a1 b2 d2^	108.6 ± 4.6^b3^
VR200	104.8 ± 4.8	139.5 ± 3.9^a2^	108.3 ± 5.4^b2 c2 d1^	102.4 ± 4.8^b3^
VR400	102.6 ± 5.5	137.1 ± 3.7^a2^	106.8 ± 4.4^b2 c2 d1^	99.9 ± 5.3^b3^
DS	100.8 ± 5.7	134.7 ± 2.9^a2^	98.9 ± 6.1^b3 c3^	97.3 ± 4.2^b3 c3^

Values are expressed in mean ± standard error. NC, normal control that receives 10 ml/kg normal saline; VR100, treatment group that receives *V. radiata* extract of 100 mg/kg; VR200, treatment group that receives *V. radiata* extract of 200 mg/kg; VR400, treatment group that receives *V. radiata* extract of 400 mg/kg; DS, treatment group that receives glibenclamide (5 mg/kg); ^a^ compared to BGL at 0 min; ^b^ compared with BGL after 30 min; ^c^ compared with NC; ^d^ compared with DS;^1^*P* < 0.05,^2^*P* < 0.01,^3^*P* < 0.001. BGL, blood glucose levels. Time refers to the time after extract administration.

**Table 3:** Antihyperglycemic activity of *V. radiata* on alloxan-induced diabetic mice.

Group (*n* = 6)	BGL in mg/dl		
	Baseline	7^th^ day	14^th^ day
DC	231.6 ± 17.2^n3^	222.5 ± 11.7^n3^	215.3 ± 13.1^n3^
VR100	242.2 ± 19.4^n3^	214.3 ± 15.8^*β*3 n3^	197.5 ± 21.5^a2 *β*3 n3^
VR200	235.9 ± 17.3^n3^	211.7 ± 13.5^a1^^*β*3 n3^	180.2 ± 15.8^a3 *β*3 n3^
VR400	240.8 ± 23.1^n3^	207.2 ± 12.5^a1 *β*3 n3^	172.4 ± 9.8^a3 *β*3 n3^
DS	247.6 ± 20.5^n3^	191 ± 18.2^a2 *β*3 n3^	150.9 ± 8.7^a3 *β*3 n3^
NC	104.3 ± 9.7	98.4 ± 10.2	89.7 ± 7.8

Values are expressed in mean ± standard error. DC, diabetic control that receives 10 ml/kg normal saline; NC, normal control that receives 10 ml/kg normal saline; VR100, treatment group that receives *V. radiata* extract of 100 mg/kg; VR200, treatment group that receives *V. radiata* extract of 200 mg/kg; VR400, treatment group that receives *V. radiata* extract of 400 mg/kg; DS, positive control that receives glibenclamide (5 mg/kg); ^a^ compared to DC, ^n^ compared to NC, and ^*β*^ compared to baseline BGL.^1^*P* < 0.05,^2^*P* < 0.01,^3^*P* < 0.001; BGL, blood glucose level.

**Table 4:** Effect of *V. radiata on* body weight of alloxan-induced diabetic mice.

Group (*n* = 6)	Body weight in gram			
	Before diabetes	Baseline after diabetes	7^th^ day	14^th^ day
DC	26.2 ± 3.3	24.6 ± 4.1	21.7 ± 3.8^n3^	19.5 ± 3.6^n3 *β*2^
VR100	27.5 ± 3.8	23.9 ± 2.4	25.2 ± 3.4^n1^	26.1 ± 3.9^a1 n3^
VR200	28 ± 3.5	24.9 ± 2.8	27.6 ± 2.5^n2^	27.9 ± 3.2^a3 n1^
VR400	27.9 ± 4.6	22.8 ± 2.9	28.6 ± 3.4^a1^	29.2 ± 2.3^a3^
DS	28.5 ± 3.4	24.6 ± 4.03	30.6 ± 2.2^a3^	31.4 ± 2.8^a3^
NC	26.6 ± 4.1	26.9 ± 2.7	31 ± 3.7	31.8 ± 2.9^*β*1^

Values are expressed in mean ± standard error. DC, diabetic control that receives 10 ml/kg normal saline; NC, normal control that receives 10 ml/kg normal saline; VR100, treatment group that receives *V. radiata* extract of 100 mg/kg; VR200, treatment group that receives *V. radiata* extract of 200 mg/kg; VR400, treatment group that receives *V. radiata* extract of 400 mg/kg; DS, positive control that receives glibenclamide (5 mg/kg); ^a^ compared to DC, ^n^ compared to NC, and ^*β*^ compared to baseline body weight.^1^*P* < 0.05,^2^*P* < 0.01,^3^*P* < 0.001.

**Table 5:** Effect of *V. radiata* on lipid profile in alloxan-induced diabetic mice.

Group (*n* = 6)	Serum lipid levels in mg/dl		
	Triglyceride	Total cholesterol	High-densitylipoprotein cholesterol
DC	162.8 ± 7.3^n3^	190.3 ± 3.3^n3^	21.7 ± 2.6^n3^
VR100	155.3 ± 4.4^n3^	166.6 ± 2.2^a3 n3^	27.5 ± 1.9^a1 n3^
VR200	153.5 ± 3.5^a1 n3^	170.5 ± 12.1^a3 n3^	28.1 ± 2.4^a1 n3^
VR400	150.1 ± 2.8^a3 n3^	166 ± 4.5^a3 n3^	30.8 ± 2.5^a3 n3^
DS	77.5 ± 4.5^a3 b3 c3 d3^	96 ± 2.9^a3 b3 c3 d3^	38.7 ± 5.1^a2 b3 c3 d3^
NC	73.3 ± 3.7	83.3 ± 3.7	40.2 ± 3.2

Values are expressed in mean ± standard error. DC, diabetic control that receives 10 ml/kg normal saline; NC, normal control that receives 10 ml/kg normal saline; VR100, treatment group that receives *V. radiata* extract of 100 mg/kg; VR200, treatment group that receives *V. radiata* extract of 200 mg/kg; VR400, treatment group that receives *V. radiata* extract of 400 mg/kg; DS, positive control that receives glibenclamide (5 mg/kg); ^a^ compared to DC, ^b^ compared to VR100, ^c^ compared to VR200, ^d^ compared to VR400, and ^n^ compared to NC.^1^*P* < 0.05,^2^*P* < 0.01,^3^*P* < 0.001.

## Data Availability

Access to raw data is possible upon justifiable request.

## Abbreviations

ALT: Alanine aminotransferase
ANOVA: Analysis of variance
AST: Aspartate aminotransferase
BGL: Blood glucose levels
DM: Diabetes mellitus
HDL: High-density lipoprotein cholesterol
LD50: Median lethal dose
NS: Normal saline
OECD: Organization for economic co-operation and development
SE: Standard error
SPSS: Statistical software package for social science
TC: Total cholesterol
TG: Triglyceride.

## Supplementary Materials

Supplementary Materials The ARRIVE Essential 10: author checklist.
